# Glucocorticoid Regulation of SLIT/ROBO Tumour Suppressor Genes in the Ovarian Surface Epithelium and Ovarian Cancer Cells

**DOI:** 10.1371/journal.pone.0027792

**Published:** 2011-11-23

**Authors:** Rachel E. Dickinson, K. Scott Fegan, Xia Ren, Stephen G. Hillier, W. Colin Duncan

**Affiliations:** Medical Research Council Centre for Reproductive Health, The University of Edinburgh, Edinburgh, United Kingdom; Virginia Commonwealth University, United States of America

## Abstract

The three SLIT ligands and their four ROBO receptors have fundamental roles in mammalian development by promoting apoptosis and repulsing aberrant cell migration. *SLITs* and *ROBOs* have emerged as candidate tumour suppressor genes whose expression is inhibited in a variety of epithelial tumours. We demonstrated that their expression could be negatively regulated by cortisol in normal ovarian luteal cells. We hypothesised that after ovulation the locally produced cortisol would inhibit *SLIT/ROBO* expression in the ovarian surface epithelium (OSE) to facilitate its repair and that this regulatory pathway was still present, and could be manipulated, in ovarian epithelial cancer cells. Here we examined the expression and regulation of the SLIT/ROBO pathway in OSE, ovarian cancer epithelial cells and ovarian tumour cell lines. Basal *SLIT2*, *SLIT3*, *ROBO1*, *ROBO2* and *ROBO4* expression was lower in primary cultures of ovarian cancer epithelial cells when compared to normal OSE (*P*<0.05) and in poorly differentiated SKOV-3 cells compared to the more differentiated PEO-14 cells (*P*<0.05). Cortisol reduced the expression of certain *SLITs* and *ROBOs* in normal OSE and PEO-14 cells (*P*<0.05). Furthermore blocking SLIT/ROBO activity reduced apoptosis in both PEO-14 and SKOV-3 tumour cells (*P*<0.05). Interestingly *SLIT/ROBO* expression could be increased by reducing the expression of the glucocorticoid receptor using siRNA (*P*<0.05). Overall our findings indicate that in the post-ovulatory phase one role of cortisol may be to temporarily inhibit SLIT/ROBO expression to facilitate regeneration of the OSE. Therefore this pathway may be a target to develop strategies to manipulate the SLIT/ROBO system in ovarian cancer.

## Introduction

The secreted Slit glycoprotein and its Robo receptor were originally identified as important axon guidance molecules in the developing *Drosophila* nervous system [1], [2]. Their role is evolutionary conserved as vertebrate SLIT (SLIT1, SLIT2, SLIT3) and ROBO (ROBO1, ROBO2, ROBO3, ROBO4) also inhibit aberrant neuron migration [3]. However most members of the vertebrate SLIT and ROBO families are also expressed outside of the nervous system and have been linked with the development of a variety of organs including the mammary gland and ovary [4], [5]. During organogenesis the SLIT/ROBO interaction is thought to regulate numerous processes including cell proliferation, apoptosis, adhesion and migration of non-neuronal cells [6], [7].

Molecules that have important roles in development are often dysregulated in cancer [8]. Indeed the *SLITs* and *ROBOs* are candidate tumour suppressor genes whose expression is reduced in numerous epithelial tumour cell types, mainly through deletion, loss of heterozygosity and promoter region hypermethylation [9]. This includes cancers derived from reproductive tissues including cervical, prostate and ovarian germ-line tumours [10]–[13]. Recent functional studies have also supported the theory that the SLITs and ROBOs have tumour suppressor activities. The SLIT/ROBO pathway promoted programmed cell death and/or reduced proliferation of fibrosarcoma, oesophageal, hepatocellular, colorectal, prostate and breast carcinoma cells [14]–[17]. SLIT2 also inhibited the invasion of numerous different types of tumour cells including those from the prostate, breast, endometrium and ovary [13], [18], [19].

The SLIT/ROBO pathway has now also been shown to have physiological roles in normal reproductive tissues [6]. SLIT/ROBO signalling seems to regulate placental angiogenesis and trophoblast function in an autocrine and/or paracrine manner [20]. In addition, most of the SLITs and ROBOs are also temporally regulated during the normal menstrual cycle in the endometrium and are expressed in the fallopian tube [21]. Furthermore there is increased expression of the *SLITs* and *ROBOs* in the adult corpus luteum during the late-luteal phase of the ovarian cycle. At this time the SLIT/ROBO interaction may act to promote its disintegration by stimulating apoptosis and inhibiting migration of luteal cells [22]. In the corpus luteum and endometrium expression of SLITs and ROBOs are hormonally regulated. There was reduced *SLIT/ROBO* expression in the decidualised endometrium of early pregnancy [21]. In addition the luteotrophic molecules, human chorionic gonadotrophin [23] and cortisol [24], that are increased in early pregnancy, reduce the expression of *SLITs* and *ROBOs* in luteal cells [22].

Around 90% of ovarian malignancies are classified as epithelial tumours that are thought to derive from the ovarian surface epithelium (OSE) [25]. The risk of ovarian cancer is positively correlated with the number of ovulations [26]. Thus recurrent injury and subsequent repair of the OSE during ovulation may predispose this tissue to neoplasia [27]. Ovulation is an inflammatory event disrupting the OSE, but requiring resolution. This repair is facilitated by an increased local production the anti-inflammatory steroid cortisol via up-regulation of 11ß-hydroxysteroid dehydrogenase type 1 [28].

We hypothesised that the OSE express SLITs and ROBOs and that cortisol could temporarily reduce the expression of these tumour suppressor genes to facilitate survival, proliferation and migration of these cells during the repair process. If this was the case this pathway might have a role in ovarian cancer progression and if it remains active in malignant OSE cells it may offer therapeutic strategies to manipulate these genes. We therefore investigated the expression, localisation and regulation of the SLIT/ROBO pathway in the OSE. We also examined whether the SLITs and ROBOs were aberrantly expressed and hormonally regulated in ovarian cancer cells. Furthermore we analysed the functional significance of a perturbed SLIT/ROBO pathway in ovarian cancer cells.

## Results

### The SLIT/ROBO pathway is differentially expressed in human OSE, ovarian cancer cells and ovarian tumour cell lines

**Figure 1:**
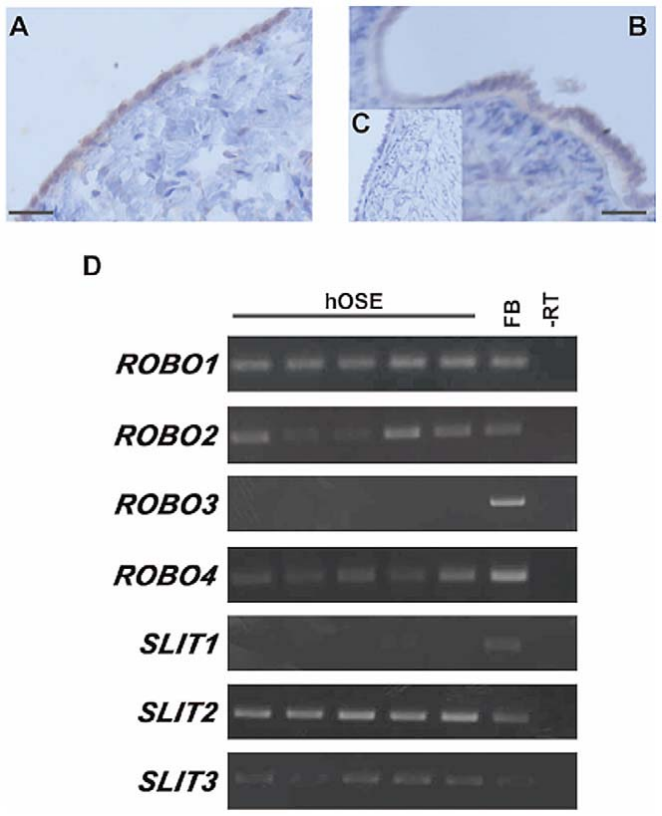
Expression analysis of the *SLIT* and *ROBO* gene families in human OSE (hOSE). **A**) Light-field microscopy of ovary showing specific staining for SLIT2 (brown) in the OSE. **B**) Likewise positive ROBO1 (brown) staining was also observed in the OSE. **C**) No staining was observed in negative controls. Scale bars represent 100 µm. **D**) RT-PCR for *SLITs* and *ROBOs* in cultured hOSE. With the exception of *SLIT1* and *ROBO3* the members of the *SLIT* and *ROBO* families were expressed in hOSE. FB = Fetal Brain positive control; -RT = RT negative negative control.

**Figure 2:**
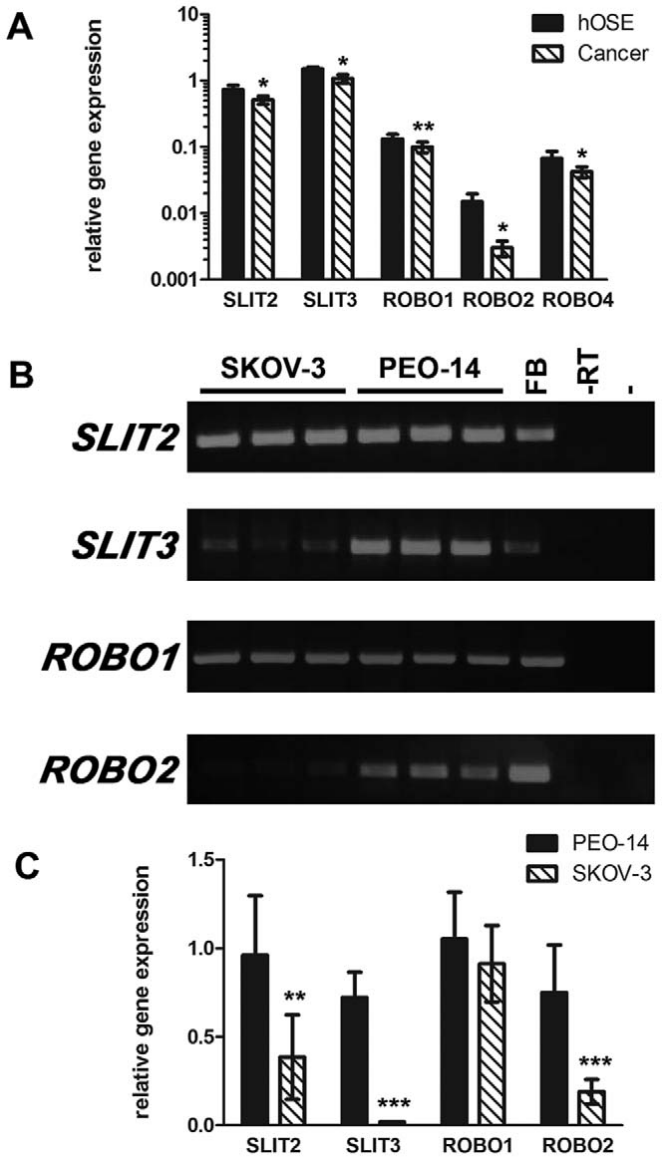
Expression of the *SLITs* and *ROBOs* in ovarian cancer. **A**) Real-time quantitative PCR showing increased *SLIT2*, *SLIT3*, *ROBO1*, *ROBO2* and *ROBO4* in primary cultures of hOSE compared to malignant epithelial cells cultured from the ascites of ovarian cancer patients. **B**) RT-PCR showing that both the SKOV-3 and PEO-14 cell lines expressed *SLIT2*, *SLIT3*, *ROBO1* and *ROBO2*. **C**) Real-time quantitative PCR showing *SLIT2*, *SLIT3* and *ROBO2* transcripts were more abundant in the well differentiated PEO-14 cells compared to the poorly differentiated SKOV-3 cells. FB = Fetal Brain positive control; -RT = RT negative negative control; - = DNA negative negative control; * = *P*<0.05; ** = *P*<0.01; *** = *P*<0.005.

SLIT2 and ROBO1 could be immunolocalised to the normal human ovarian surface epithelium (Fig. 1A–C). RT-PCR analysis confirmed the expression of *SLIT2* and *ROBO1* in primary cultures of OSE and demonstrated that there was some expression of *SLIT3*, *ROBO2* and *ROBO4* in these cells (Fig. 1D). We then investigated the expression of these genes in primary cultures of malignant cells derived from the ascitic fluid of women with epithelial ovarian cancer. *SLIT2*, *SLIT3*, *ROBO1*, *ROBO2* and *ROBO4* were also expressed in these cells but quantitative analysis showed that they were reduced by 25–82% when compared to primary cultures of normal OSE (Fig. 2A) (*P*<0.05).

In order to confirm that malignant OSE cells had a reduced expression of *SLITs* and *ROBOs* we examined their expression in two different ovarian tumour cell lines. The PEO-14 cell line is derived from a well-differentiated ovarian adenocarcinoma and has similarities with more benign ovarian epithelial cells and early stage ovarian cancer. In contrast, the SKOV-3 cell line is derived from a poorly differentiated ovarian adenocarcinoma and is more characteristic of an advanced tumour [29]. Both these cell lines expressed *SLIT2*, *SLIT3*, *ROBO1* and *ROBO2* (Fig. 2B). However, paralleling our results in the primary cell culture, *SLIT2*, *SLIT3* and *ROBO2* expression was decreased by between 58% and 97% in the poorly differentiated SKOV-3 cells when compared to the well-differentiated PEO-14 cells (Fig. 2C) (*P*<0.05). Although PEO-14 and SKOV-3 cells may differ in other aspects as well as their differentiation status these data suggest that expression of the *SLIT/ROBO* tumour suppressor gene pathway may be reduced during tumour development or progression.

### Blocking the SLIT/ROBO interaction decreases apoptosis in ovarian tumour cells

**Figure 3:**
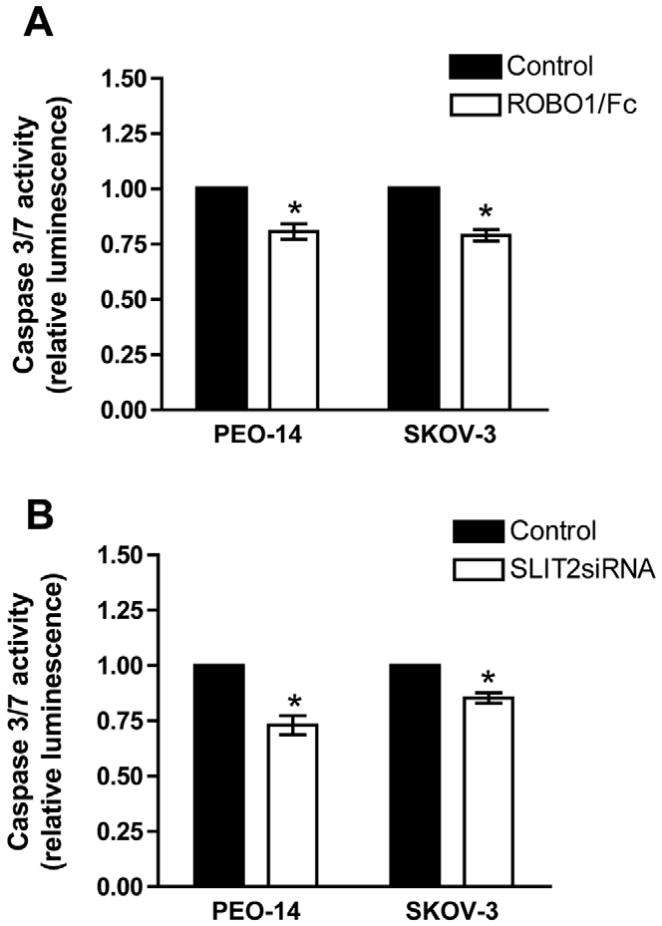
Inhibiting SLIT-ROBO signalling decreases apoptosis as measured by an activated caspase-3/7 assay. **A**) Treatment with the ROBO1/Fc chimera reduced caspase-3/7 activity, when compared to treatment PBS/0.1% (w/v) BSA (Control) in both PEO-14 and SKOV-3 cells. **B**) Transfection with *SLIT2* siRNA reduced caspase-3/7 activity, when compared to treatment with a scrambled siRNA control. * = *P*<0.05.

The effect of the SLIT/ROBO pathway on cell survival was investigated using a recombinant ROBO1/Fc chimera, which acts as a ligand trap to inhibit the SLIT/ROBO interaction, and direct inhibition of *SLIT2* using siRNA. Treatment of PEO-14 and SKOV-3 cells with the ROBO1/Fc chimera did not affect cell proliferation (*P*>0.05, data not shown). However in both the PEO-14 and SKOV-3 cells blocking SLIT action with the ROBO1/Fc chimera reduced apoptosis by 20–21% as measured by an activated caspase-3/7 assay (*P*<0.05) (Fig. 3A). Transient transfection of *SLIT2* siRNA reduced solely *SLIT2* expression in both PEO-14 and SKOV-3 cells. PEO-14 and SKOV-3 cells with reduced *SLIT2* expression had a significant 17–26% decrease in cleaved Caspase-3 and -7 activities (*P*<0.05, paired t-test) (Fig. 3B). This suggests that a reduction in the SLIT/ROBO gene pathway is associated with increased cell survival.

### Cortisol negatively regulates the expression of *SLITs* and *ROBOs* in OSE and a well-differentiated ovarian cancer cell line

**Figure 4:**
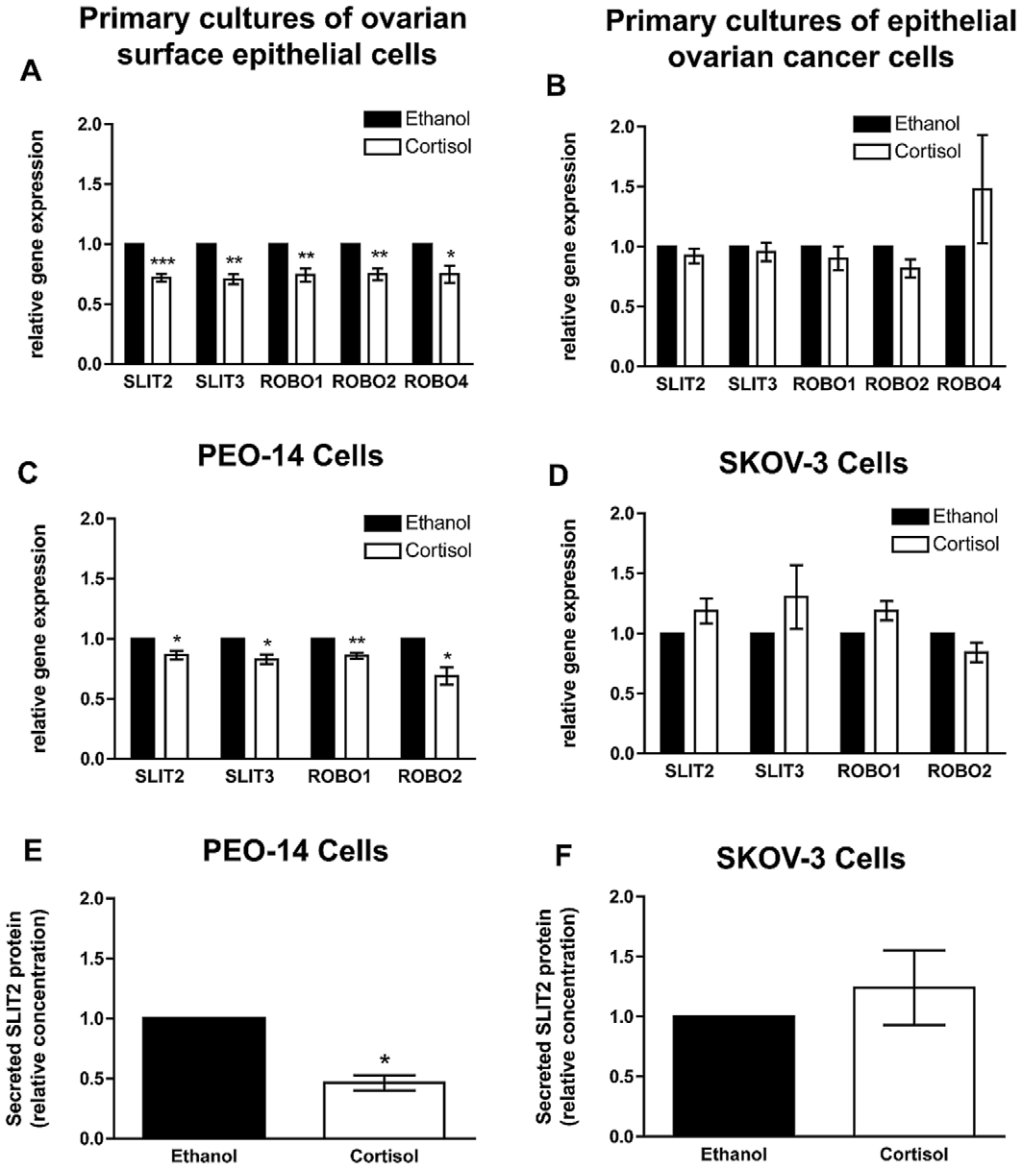
The effect of cortisol on the SLIT/ROBO pathway. **A**) Real-time quantitative PCR showing that Cortisol (1000 nM), compared to Ethanol carrier control, reduced *SLIT2*, *SLIT3*, *ROBO1*, *ROBO2* and *ROBO4* expression in primary cultures of hOSE. **B**) Cortisol did not alter the expression of *SLITs* and *ROBOs* in primary cultures of ovarian epithelial cancer cells. **C**) *SLIT2*, *SLIT3*, *ROBO1* and *ROBO2* expression was significantly reduced by Cortisol in more differentiated PEO-14 cells. **D**) However expression of these *SLITs* and *ROBOs* was not regulated by Cortisol in the poorly differentiated SKOV-3 cells. **E**) Cortisol reduced secreted levels of SLIT2 protein in the PEO-14 cells (control secretion is 0.5 ng/ml). **F**) Cortisol did not affect secreted levels of SLIT2 protein in the SKOV-3 cells (control secretion is 0.5 ng/ml). * = *P*<0.05; ** = *P*<0.01.

We then investigated whether the expression of the SLIT/ROBO pathway in ovarian epithelial cells was regulated. In human ovarian luteal cells the SLIT/ROBO pathway could be physiologically inhibited by cortisol [22]. As cortisol is produced locally in the OSE, and has an anti-inflammatory role after ovulation [30], we examined whether cortisol could regulate SLIT/ROBO expression in the OSE. *SLIT2*, *SLIT3*, *ROBO1*, *ROBO2* and *ROBO4* expression was reduced by 25–30% in cortisol (1000 nM) treated primary cultures of normal OSE (*P*<0.05) (Fig. 4A). However, in primary cultures of malignant epithelial cells cortisol did not result in any further reduction in the expression of these genes (*P*>0.05) (Fig. 4B).

**Figure 5:**
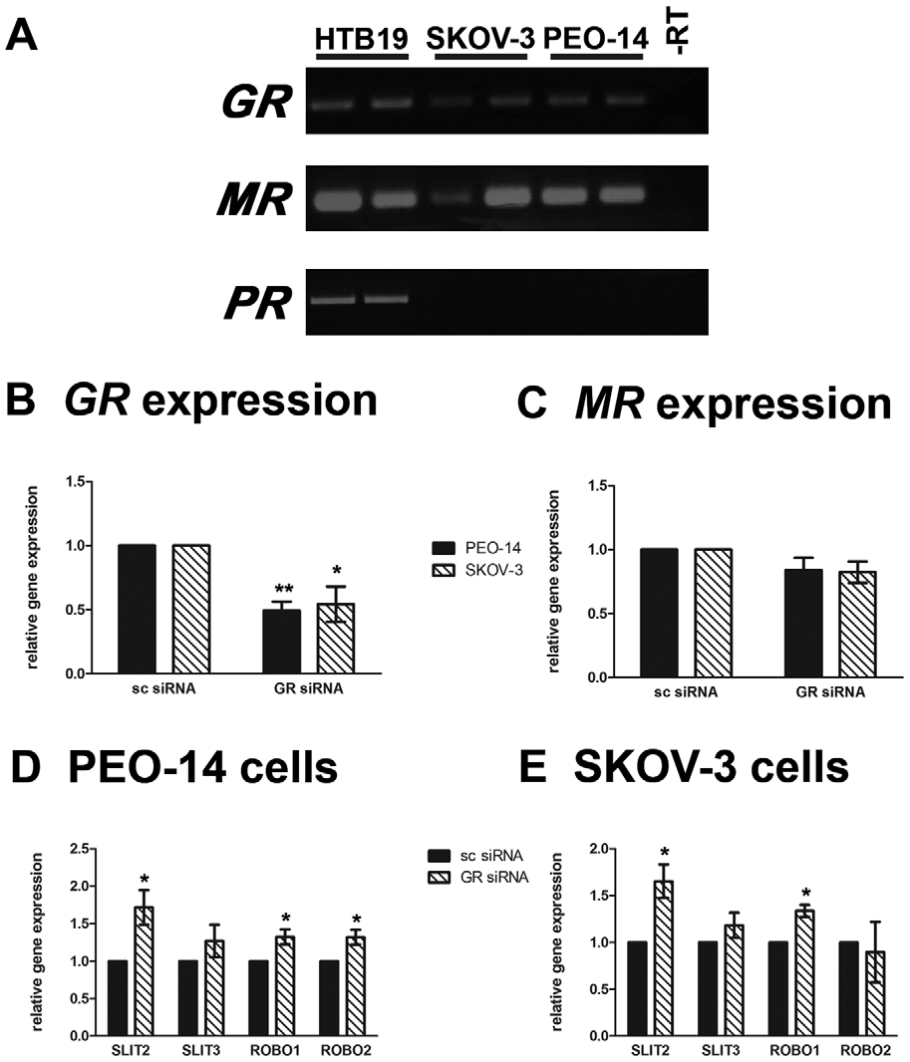
The effect of manipulation of *GR* on *SLITs* and *ROBOs* in ovarian cancer cells. **A**) RT-PCR showing that PEO-14 and SKOV-3 cells expressed *GR* and *MR* but not *PR*. The breast tumour cell line HTB-19 was used as a positive control and –RT was used as a negative control. **B**) Real-time quantitative PCR demonstrating that transfection with the *GR* siRNA reduced *GR* expression in both cell lines when compared to the scrambled (sc) siRNA control. **C**) Confirmation that *GR*siRNA did not affect *MR* expression in PEO-14 and SKOV-3 cells. **D**) Quantitative Real-time PCR showing an increase in *SLIT2*, *ROBO1* and *ROBO2* expression after *GR* knock down by *GR*siRNA in PEO-14 cells. **E**) Demonstration that *GR*siRNA transfected SKOV-3 cells also had increased *SLIT2* and *ROBO1* expression. * = *P*<0.05.

The PEO-14 and SKOV-3 ovarian tumour cell lines also have the potential to respond to cortisol treatment as they express the glucocorticoid receptor (GR). In addition they express the mineralocorticoid receptor (MR) but do not express the progesterone receptor (PR) (Fig. 5A). In the more differentiated PEO-14 cells, cortisol reduced the expression of *SLIT2*, *SLIT3*, *ROBO1* and *ROBO2* by 13–31% (*P*<0.05) (Fig. 4C). Furthermore cortisol treatment reduced secreted SLIT2 protein concentration by 53% (*P*<0.05) (Fig. 4E). Like the primary cell cultures, the regulation of these *SLITs* and *ROBOs*, as well as the secreted SLIT2 protein, was lost in the more malignant, and less differentiated, SKOV-3 cells (*P*>0.05) (Fig. 4D,F). This suggests that cortisol may have a physiological role in reducing the SLIT/ROBO interaction during repair of the OSE and that this pathway may still be active in some early stage ovarian cancers.

### Manipulation of *SLITs* and *ROBOs* in ovarian tumour cells by targeting the glucocorticoid receptor

Thus, although glucocorticoids have a theoretical detrimental effect on early ovarian cancer cells, it means that manipulation of the GR, with the aim of increasing *SLIT/ROBO* gene expression, is a potential therapeutic target. We therefore “knocked down” *GR* using *GR* siRNA in cortisol-responsive PEO-14 cells. Transfection of the *GR*siRNA for 48 hours caused a significant 63% reduction in *GR* expression (*P*<0.001) (Fig. 5B) without influencing *MR* expression (Fig. 5C). This increased the expression of the *SLIT2*, *ROBO1* and *ROBO2* tumour suppressor genes (*P*<0.05) (Fig. 5D). In addition, transfection of the *GR* siRNA resulted in a similar reduction in *GR* expression in the cortisol-unresponsive SKOV-3 cells (*P*<0.01) (Fig. 5B). Importantly the expression of the *SLIT2* and *ROBO1* tumour suppressor genes was also enhanced by *GR*siRNA transfection (*P*<0.05) (Fig. 5E).

PEO-14 and SKOV-3 cells were also treated with mifepristone (RU486), which functions as a GR antagonist. RU486 treatment alone did not influence the expression of *SLIT2*, *SLIT3*, *ROBO1* or *ROBO2* in either cell line (*P*>0.05, data not shown). However RU486 treatment did abolish the cortisol mediated negative regulation of *SLIT/ROBO* expression in PEO-14 cells (data not shown). This suggests that there may be ligand independent effects of GR on SLIT/ROBO expression and confirms that even in poorly differentiated cancer cells manipulation of GR can regulate the expression of tumour suppressor genes.

## Discussion

In this study we established that the normal human adult OSE expresses, at the RNA level, *SLIT2*, *SLIT3*, *ROBO1*, *ROBO2* and *ROBO4*. Using immunohistochemistry we also showed that SLIT2 and ROBO1 are also expressed at the protein level. As each of the SLITs is able to interact with each of the ROBOs, with the possible exception of ROBO4, it is likely that the SLIT/ROBO interaction is occurring in the OSE. This is not surprising as these molecules are expressed in other ovarian cells including the granulosa lutein, theca lutein and luteal fibroblasts cells of the adult corpus luteum [22] and the pre-granulosa cells and oocytes of primordial follicles within the developing fetal ovary [5]. The normal ovary is therefore a site of the physiological autocrine or paracrine actions of the SLIT/ROBO system.

We found that in normal OSE the SLIT/ROBO system can be regulated by cortisol. Cortisol reduced the expression of *SLIT2*, *SLIT3*, *ROBO1*, *ROBO2* and *ROBO4* in primary cultures of OSE cells. We have previously shown that the same concentration of cortisol can inhibit the expression of *SLIT2* and *SLIT3* in primary cultures of luteinised granulosa cells and luteal fibroblast-like cells [22]. After ovulation there is an increase in the local production of cortisol in the OSE that may act to encourage tissue repair and renewal [28]. Over the range of physiologically relevant concentrations in OSE cells cortisol has been shown to have an anti-inflammatory action and can block interleukin-1 stimulated MMP-9 expression [30], [31]. In addition we have previously shown that cortisol, by negatively regulating the expression of SLITs and ROBOs, inhibits apoptosis and facilitates cell migration [22]. This implies that after ovulation one of the effects of locally produced cortisol may be to temporarily reduce the expression of the SLIT/ROBO tumour suppressor genes to facilitate repair of the damaged OSE.

In many epithelial cancers there is an associated loss of the expression of members of the SLIT/ROBO family [9]. For example decreased expression of *SLIT* and *ROBO* transcripts has been observed in oesophageal squamous cell, hepatocellular, lung, prostate and breast carcinoma [10], [15], [16], [32], [33]. This reduction in expression however is not universal and some cancers, such as gliomas [34] and recurrent endometrial cancer [35] maintain or increase the SLIT/ROBO pathway. However alterations in the expression of this pathway in malignant epithelial cells from ovarian cancer has not previously been studied.

We cultured malignant epithelial cells from the ascitic fluid of patients with advanced epithelial ovarian cancer and compared SLIT/ROBO expression to that in normal OSE. We found reduced expression of *SLIT2*, *SLIT3*, *ROBO1*, *ROBO2* and *ROBO4* in malignant cells. Although we believe these cultures are pure cultures of malignant ovarian cells [36], it is possible that there may be non-malignant contaminating cells in some cultures. We therefore also compared the well-differentiated PEO-14 cells with the poorly differentiated SKOV-3 cells. In both cases the more malignant cells had lower expression of the SLITs and some of the ROBOs. It is therefore likely that ovarian cancer can be added to the list of tumours with reduced SLIT/ROBO expression.

We went on to investigate what effects a less active SLIT/ROBO pathway may have on cell function. Recent studies have implied that SLIT2 can inhibit the invasion of endometrial and ovarian tumour cell lines [19]. We have also shown that inhibition of SLIT/ROBO signalling in primary cultures luteal fibroblasts promoted cell migration [22]. In addition blocking SLIT action in luteal cells from the normal ovary inhibited apoptosis and reduced in Caspase-3/7 activity [22]. There was a reduction in Caspase-3/7 mediated apoptosis in PEO-14 and SKOV-3 cells when SLIT/ROBO signalling was inhibited by blocking SLIT activity using a ROBO1/Fc chimera and SLIT2 synthesis using siRNA technology. Increased apoptosis, associated with reduced expression of the Bcl-2 and Bcl-xl anti-apoptotic molecules, was seen in SLIT2 transfected fibrosarcomas and oesophageal squamous cell carcinomas [16]. SLIT2 could also induce apoptosis associated with activation of Caspase 3 in breast and lung tumour cell lines [37]. Overall a reduction in SLIT/ROBO activity is associated with increased cell survival and migration and this is likely to be relevant in ovarian cancer and its progression.

If the glucocorticoid-mediated inhibition of the SLIT/ROBO pathway is still manifested in malignant epithelial cells in ovarian cancer then cortisol may have effects on cell survival and migration that would be detrimental to the patient. In our primary cultures of advanced ovarian cancer, as well as the poorly differentiated SKOV-3 ovarian cancer cell line, the regulation of SLITs and ROBOs by cortisol was lost. However it was maintained in the more differentiated PEO-14 tumour cell line. This implies that the pathway may still be active in the early stages of ovarian cancer or in less malignant phenotypes. Interestingly glucocorticoids could inhibit apoptosis during fibrosarcoma development [38] and in ovarian tumour cell lines and cells from the ascitic fluid of ovarian cancer patients [39]. Dexamethasone can also curtail apoptosis induced by chemotherapy in a variety of different tumour types including breast, prostate, cervical and ovarian carcinoma cells [40]–[42]. Therefore glucocorticoid inhibition of SLITs and ROBOs might still be possible in some malignant cells.

As the up-regulation of SLITs has been shown to have inhibitory effects on tumour growth and invasion it is possible that manipulation of the glucocorticoid pathway has therapeutic utility. RU486, a GR and PR antagonist, can induce apoptosis in prostate and ovarian cancer cells [43], [44]. Blockade of cortisol activity in PEO-14 and SKOV-3 cells, which lack PR, using RU486 did not influence the expression of *SLITs* and *ROBOs* in either cell line. However RU486 did abolish the negative regulation of *SLIT/ROBO* expression in PEO-14 cells.

More importantly when we inhibited endogenous *GR* expression, using siRNA, there was an increase in the expression of certain *SLITs* and *ROBOs* in both PEO-14 and SKOV-3 cells. This implies that the *SLITs* and *ROBOs* could be regulated, at the transcriptional level, by GR and that a major role for GR in controlling *SLIT/ROBO* expression may be ligand independent. Our bioinformatic analysis revealed several GR-responsive and related elements (GREs) in the promoter regions of *SLIT2*, *SLIT3*, *ROBO1*, *ROBO2* and *ROBO4*. Intriguingly, in neuroblastoma cells, the activated GR directly interacts with p53 and inhibits p53 dependent cell cycle arrest and apoptosis [45]. However GR can act as a tumour suppressor in other types of tumours including skin cancer [46]. Therefore the exact function of GR in cancer could be dependent on the tumour type.

In summary, this study has provided further evidence that the SLIT/ROBO pathway has a role in normal ovarian physiology and is regulated by hormones including glucocorticoids. We have also shown that the SLITs and ROBOs seem to be aberrantly expressed in ovarian cancer. Furthermore reduction of this pathway could augment tumourigenesis and progression through a dysregulation of cellular processes including apoptosis and repulsion of cell migration. Overall these data support the concept that reduction of the SLIT/ROBO pathway is important in malignant development and progression. This research also suggests that targeting their physiological regulation by steroids may have utility in ovarian cancer.

## Materials and Methods

### Cell and tissue collection

All cells and tissues were obtained with informed written consent after approval from the Lothian Medical Research Ethics Committee. Human OSE cells were obtained from the ovaries of premenopausal women (n = 5) undergoing elective surgery for non-malignant gynaecological conditions as described previously [29]. Malignant epithelial cells were obtained from the ascitic fluid of women (n = 8) having surgery for advanced epithelial ovarian cancer as described previously [36]. Normal human ovarian tissue had been collected for a complimentary study [47]. The SKOV-3 and PEO-14 cell lines were kindly provided by P. Pujol, INSERM, Montpellier, France and S. Langdon, University of Edinburgh, Edinburgh, UK.

### Cell culture and treatments

Primary OSE cells and primary ovarian cancer cells were routinely maintained in culture media consisting of Medium 199 (Invitrogen, Paisley, UK) and MCDB105 (Sigma-Aldrich Corp., Gillingham, UK) (pH 7.3, 1∶1 vv^−1^) supplemented with 15% (v/v) Fetal Bovine Serum, 50 IU/ml Penicillin, 50 µg/ml Streptomycin and 2 mM L-Glutamine. PEO-14 and SKOV-3 cells were cultured in the same media however it was supplemented with 10% (v/v) FBS. For the cortisol treatment studies cells were seeded in six-well plates at a density of 2×10^5^ cells/well. After 24 hours fresh media containing either 1000 nM Cortisol [22], [30], [31] in ethanol with or the equivalent volume of ethanol (0.001% v/v) was added to the cells. In other experiments 50 µM RU486 [24] (Sigma) with and without cortisol was used. Each treatment was carried out in technical triplicate. After 24 hours 1 ml of media was removed from each well and stored at −20°C for the enzyme-linked immunosorbent assay (ELISA) experiment and RNA was extracted from the cells as described below.

For the ROBO1/Fc treatment studies cells were seeded at 2×10^4^ cells/well in 96-well plates. After 24 hours fresh media containing either recombinant rat ROBO1/Fc chimera (R&D Systems, Inc., Abingdon, UK; 1 µg/ml) or the equivalent volume of PBS/0.1% (w/v) BSA was added to the cells. Treatments were carried out in technical quadruplicate. Forty-eight hours later the cells were analysed for apoptosis using the Caspase-Glo 3/7 assay as described below.

### Short interfering RNA technology

Twenty-four hours before transfection PEO-14 or SKOV-3 cells were seeded in antibiotic free media so that cells were 50% confluent at the time of transfection. Short interfering RNA oligonucleotides against GR and SLIT2 as well as a negative controls, with no significant sequence similarity to human gene sequences, were obtained from Applied Biosystems (Warrington, UK). They were transiently transfected into the cells using Oligofectamine transfection reagent according to manufacturers' instructions (Invitrogen). Briefly, the siRNA oligonucleotides were diluted to a final concentration of 200 nM in Opti-MEM I Reduced Serum Media (Invitrogen) and combined with diluted Oligofectamine to allow the siRNA∶Oligofectamine complexes to form at room temperature. The siRNA∶Oligofectamine complex was then added dropwise to each well and the cells were then incubated at 37°C, 5% CO_2_ for 48–72 hours. For expression analysis experiments the cells were grown in 6-well plates and each transfection was performed in triplicate. For the caspase-3/7 activity assay experiments the cells were grown in 96-well plates and each transfection was performed in quadruplicate.

### Expression analysis

RNA was extracted from each well of the cells using the RNeasy Mini kit (QIAGEN Ltd., Crawley, UK) and treated with deoxyribonuclease I (QIAGEN). RNA was used as a template for cDNA synthesis using Taqman reverse transcriptase reagents (Applied Biosystems). Primers specific for the *SLITs* and *ROBOs* have been previously described in detail [22]. PCR was performed on an Eppendorf Mastercycler gradient authorised thermocycler (PerkinElmer, Inc., Waltham, MA) using GoTaq Flexi DNA polymerase (Promega Ltd., Southampton, UK). The PCR thermocycle consisted of an initial denaturation of 5 min at 95°C followed by 35 cycles of 95°C for 30 sec, annealing temperature for 30 sec, 72°C for 30 sec, and a final extension of 10 min at 72°C. PCR products were visualised on a 2% (w/v) agarose gel with added ethidium bromide.

### Real-time quantitative PCR

RNA was extracted and reverse transcribed as described above. A standard curve was generated with serial dilutions of cDNA synthesised from human fetal brain total RNA (Stratagene Europe, Amsterdam, The Netherlands). Real-time PCR amplification was then performed in duplicate 10 µl reactions using *Power* SYBR Green PCR master mix (Applied Biosystems) following the manufacturer's instructions and using the ABI 7500HT fast real-time PCR system instrument (Applied Biosystems). Primers used were the same as for the expression analysis and have been described previously [22]. The ABI instrument's default settings were used for the cycling program and the melting curve analysis.

The ABI analysis software calculated quantitative values for each sample by comparing the sample threshold cycle number, where the increase in the signal associated with exponential growth of PCR products begins to be detected, to the standard curve, according to the manufacturer's manuals. In all cases the level of gene expression within the samples lay within the boundaries of the corresponding standard curve. Since the precise quality and amount of cDNA that was added to each reaction mix was difficult to assess, transcripts of glucose-6-phosphate dehydrogenase (G6PDH), a housekeeping gene, were also quantified for each sample as described above. This gene is not regulated in the samples under investigation and therefore acted as an endogenous control. Each sample was normalised on the basis of its G6PDH content by dividing the amount of target gene by the amount of housekeeping gene.

### SLIT2 ELISA

Since SLIT2 is a secreted protein, the concentration of this protein from PEO-14 and SKOV-3 cells was assessed using culture media from these experiments. Human SLIT2 protein concentration was determined quantitatively using an immunoassay kit (USCN Life Science & Technology Co., Wuhan, China). Firstly, frozen media was concentrated by freeze-drying and the dried media was reconstituted in 220 µl sample diluent buffer that was provided with the immunoassay kit. One hundred microlitres of the experimental samples and standards were loaded onto the microtitre plate in duplicate. The protocol was followed according to the manufacturers' instructions and optical density was measured using a Multiskan EX microplate photometer (Thermo Fisher Scientific Inc., Basingstoke, UK) at 450 nm. A standard curve was generated and sample concentrations were calculated using AssayZap computer software (Biosoft, Cambridge, UK).

### Caspase-3 and -7 activity assay

To measure caspase-3 and -7 activities in PEO-14 and SKOV-3 cells, the Caspase-Glo 3/7 assay was followed according to the manufacturer's instructions (Promega UK Ltd., Southampton, UK) as described previously [22]. Briefly, Caspase-Glo 3/7 reagent was added directly to the cells in culture medium in a 1∶1 ratio. The well contents were then mixed and incubated at room temperature for up to 3 hours. This resulted in cell lysis, followed by caspase cleavage of the substrate and generation of a glowtype luminescent signal, produced by luciferase. Luminescence was measured using a FLUOstar OPTIMA microplate reader (BMG Labtech Ltd., Aylesbury, UK). Luminescence was directly proportional to the amount of caspase activity present. The value for the no cell control was subtracted from the experimental values and the experiment was carried out three times to reduce the possible effects of biological variability.

### Immunohistochemistry

To investigate the localisation of ROBO1 and SLIT2 proteins, 5 µm paraffin tissue sections of normal human ovary prepared on poly-L-lysine-coated microscope slides were examined. The immunohistochemistry protocol and antibodies and controls used have been previously described in detail [5].

### Statistical analysis

Statistical analysis was conducted using a Prism software package (GraphPad Software Inc., La Jolla, CA, USA) with significance defined as *P*<0.05. After confirmation of normal distribution of samples they were analysed using either a paired or unpaired t-test as appropriate.
